# Congenital Human Cytomegalovirus Infection: A Narrative Review of Maternal Immune Response and Diagnosis in View of the Development of a Vaccine and Prevention of Primary and Non-Primary Infections in Pregnancy

**DOI:** 10.3390/microorganisms9081749

**Published:** 2021-08-16

**Authors:** Giuseppe Gerna, Chiara Fornara, Milena Furione, Daniele Lilleri

**Affiliations:** 1 Laboratories of Genetics, Transplantology and Cardiovascular Diseases, Fondazione IRCCS Policlinico San Matteo, 27100 Pavia, Italy; 2 Molecular Virology Unit, Microbiology and Virology Department, Fondazione IRCCS Policlinico San Matteo, 27100 Pavia, Italy; m.furione@smatteo.pv.it (M.F.); d.lilleri@smatteo.pv.it (D.L.)

**Keywords:** congenital cytomegalovirus, epidemiology, clinical symptoms, maternal immune response, diagnosis, prevention, management, antiviral therapy

## Abstract

Congenital cytomegalovirus infection (cCMV) may affect about 1% of all newborns all over the world as a result of either a primary or recurrent human cytomegalovirus (HCMV) infection. While about 90% of infants affected by cCMV are asymptomatic at birth, the remaining 10% are symptomatic often with neurodevelopmental impairment and sensorineural hearing loss. In view of identifying the best approach to vaccine prevention of cCMV, this review will examine the most important steps made in the study of the immune response to, and diagnosis of, HCMV infection. The maternal immune response and immune correlates of protection are being partially identified with a partial contribution given by our laboratory. The diagnosis of primary infection is often difficult to achieve in the first three months of pregnancy, which is the time primarily involved in virus transmission to the fetus in association with the most severe symptoms and *sequelae*. Prevention of cCMV is anticipated by prevention of primary infection in early pregnancy by means of different measures, such as (i) behavioral-educational measures, (ii) immunoglobulin administration, (iii) antiviral treatment with valaciclovir. However, the most promising approach to cCMV prevention appears to be the development of a non-living vaccine, including at least three viral antigens: gB, pentamer complex gHgLpUL128L, and pp65, which have been shown to be able to stimulate both the humoral and the cellular arms of the maternal immune response. Primary HCMV infection may be managed in pregnancy by counseling of the couples involved by a team of specialists that includes virologists, obstetricians, infectivologists and neonatologists.

## 1. Introduction

Human cytomegalovirus (HCMV) or human herpesvirus-5 is a human herpesvirus that is widespread in all five continents and is generally responsible for asymptomatic or mildly symptomatic infections in an immunocompetent host but may cause severe multi-organ disease in an immunocompromised host, such as HIV-1-infected patients and solid-organ/hemopoietic stem cell-transplanted patients. In the immunocompetent population, the most severe outcome of HCMV infection may occur in pregnant women with HCMV primary infection (PI) or even with non-primary infection (NPI) (reactivation or re-infection). The result of HCMV infection acquired during pregnancy may be HCMV transmission to the fetus in about 40% of the cases of PI. In cases of NPI of a seroimmune pregnant woman with a different HCMV strain, the incidence of congenital HCMV (cCMV) infection may be lower, similar or even higher. Newborns affected by cCMV will be mostly asymptomatic at birth, but could present with more or less severe *sequelae* in the following months/years. In this review, we will briefly discuss some aspects related to the epidemiology, diagnosis, the maternal immune response and the immune correlates of protection against virus transmission to the fetus in view of identifying the most effective approach to vaccine prevention of cCMV. Since our group in Pavia, Italy, has been involved in the study of cCMV for almost 40 years, an important part of the findings reported in this review originates from our daily research and clinical findings.

## 2. Epidemiology

In a recent meta-analysis, including 17 different studies, the pooled rates of vertical HCMV transmission in 10 studies on 2942 fetuses at the preconception time, periconception time, and first, second, and third trimester of pregnancy were 5.5%, 21.0%, 36.8%, 40.3%, and 66.2%, respectively. In addition, the pooled rates of fetal damage in 796 fetuses from the 10 studies at the same time were 28.8%, 19.3%, 0.9%, and 0.4% at the periconception, first, second and third trimester of pregnancy, respectively. These findings allowed the authors to conclude that, while the cCMV rate increased with the progression of pregnancy, the rate of severe fetal pathology was very rare after maternal PI occurring in the 2nd–3rd trimester of pregnancy [1,2].

While in the decades immediately following HCMV isolation (1956) it was generally reported that cCMV occurred only in HCMV-seronegative pregnant women with PI, more recently several reports have described cases of cCMV in seroimmune women. Importantly, starting in the 1980s, it was observed that there was a direct correlation between the rate of cCMV and the rate of HCMV seroprevalence [3,4,5]. While in Western Europe, the USA and Australia, the HCMV seroprevalence of women in childbearing age is about 50%, in South America, Asia and Africa the rate of seroprevalence is nearly 100% [6]. As a result, maternal NPI has been calculated to be responsible for two-thirds to three-quarters of cCMV cases in highly seropositive populations [5]. In parallel, in the USA, three-quarters of cCMV infection have been considered as due to NPI [5], while the aliquot of cCMV cases in developing countries may be even higher [7].

The prevalence of cCMV infections reaches 0.5% to 0.7% in developed countries and 1–2% in developing countries [8]. Approximately 13% of newborns with cCMV are symptomatic at birth, and the prevalence of newborns with permanent *sequelae* reaches about 20%, including 13% of newborns symptomatic at birth plus an additional 7% of newborns asymptomatic at birth [9]. Among symptoms present at birth in infants with cCMV following maternal PI in pregnancy, the most common are: intrauterine growth retardation, jaundice, hepatosplenomegaly, thrombocytopenia, purpura, microcephaly, seizures and chorioretinitis [9]. Among permanent *sequelae*, the most common are sensorineural hearing loss (SNHL), which is more frequent in symptomatic cCMV infections [10], as well as permanent intellectual and physical disabilities [11]. While for some time pre-pregnancy maternal immunity was considered to be associated with a lower rate of severe permanent *sequelae*, other reports have shown that the frequency was comparable in infants with cCMV born to mothers with PI or NPI [7,12,13,14].

## 3. Clinical Features of cCMV

Recently, the International Congenital Cytomegalovirus Recommendations Group tried to classify the different levels of severity of cCMV [15] based on clinical symptoms reported for symptomatic cCMV infections. These symptoms included: (i) a wide range of clinical manifestations, such as thrombocytopenia, hepatosplenomegaly and intrauterine growth restriction; (ii) central nervous system (CNS) involvement, as revealed by microcephaly, neuroimaging alterations, such as ventriculomegaly, cerebral calcifications, periventricular echogenicity and seizures [16]; (iii) ocular pathology, such as chorioretinitis or optic atrophy [17]. Thus, in view of providing guidelines for antiviral treatment, cCMV infections were classified as: (i) moderately to severely symptomatic, when referring to multiple clinical manifestations, or (ii) mildly symptomatic, when including just one or two symptoms or syndromes [15].

About half of infants with symptomatic cCMV infection will have permanent *sequelae*: mostly SNHL, followed by cognitive deficits, chorioretinitis and cerebral palsy [9,16]. On the other hand, infants with asymptomatic cCMV infections rarely suffered from neurodevelopmental *sequelae* and showed no difference in their intelligence quotient (IQ) compared to normal control infants [18]. SNHL has been reported as the most frequent *sequela* in both symptomatic and asymptomatic cCMV [19]. In order to detect early SNHL, newborn hearing screening was proposed instead of universal HCMV screening [20]. However, a large multicenter study showed that >40% of the infants with cCMV-associated SNHL would have been missed by the targeted hearing screening approach [21]. In addition, universal newborn screening was shown to identify all infants at risk for SNHL and also to be cost-effective [22]. In symptomatic infants with cCMV both clinical markers (microcephaly, seizures) and neuroimaging (intracerebral calcifications, ventriculomegaly) have been reported to be a good predictor of SNHL as well as adverse cognitive outcomes [16,23,24,25]. The prognostic role of HCMV viral load in predicting SNHL remains to be defined [19]. SNHL has been reported as the result of maternal PI in all three trimesters of pregnancy, but severe auditory and neurologic *sequelae* appear limited to cases acquired during the first trimester of pregnancy [2]. However, NPI appears associated to the majority of cases of cCMV throughout the world [26]. Currently, neither single strains of HCMV with different genomes nor mixed HCMV infections have been found to correlate with cCMV.

## 4. Maternal Immune Response and Immune Correlates of Protection

### 4.1. Humoral Immunity

In recent years, our group has been repeatedly interested in studying the humoral immune response to primary HCMV infection in pregnant vs. non-pregnant women, with the aim of identifying immune correlates of protection against HCMV vertical transmission to the fetus [27]. Currently, no significant difference in the peak level of IgG and IgM antibodies to crude HCMV antigen was found between the group of women transmitting (T) the virus to the fetus and the group of non-transmitting (NT) women. The only difference observed was the one relevant to the calculation of the avidity index (AI) [28] between a serum sample collected early (T1, median 31 days) and one collected late (T2, 136 days) after infection onset. The main result shown by this experiment conducted on 69 pregnant women documented the presence of two major patterns of the avidity maturation: the H (high) pattern showing a progressive increase of the AI in the presence of urea, and the L (low) pattern with a substantially unchanged or slowly decreasing AI in the presence of urea over time. Thus, the H pattern exhibited a substantial increase in AI between the two time points, whereas the L pattern did not. Therefore, the most important result was that vertical transmission was significantly higher in women with the H pattern compared to women with the L pattern. In other words, pregnant women with slow maturation of the IgG AI appeared less susceptible to vertical transmission.

Three major glycoprotein complexes (gCs) have been shown on the envelope of HCMV virions: gCI, gCII, and gCIII. UL55-encoded gB (gCI) was considered in the 1990s as the major target of the neutralizing antibody response during natural infection. On this basis, a gB-based vaccine was formulated with the intent of preventing congenital infection in pregnant women with primary HCMV infection [29] as well as HCMV infection in transplant recipients [30]. Subsequently, the gM/gN (UL100-encoded gM and UL73-encoded gN) gCII was found to be an important target of the strain-specific neutralizing antibody (NAb) response [31,32]. Finally, the gHgL (the UL75-encoded gH and the UL115-encoded gL) gCIII was found to be associated with either the UL74-encoded gO, thus giving rise to the trimeric complex (TC) gHgLgO [33], or the UL131-128 locus (pUL128L), thus giving rise to the pentameric complex (PC) gHgLpUL128L [34,35]. TC binds to its receptor, the platelet-derived growth factor receptor-α (PDGFR-α), thus mediating virus entry into human embryonic lung fibroblasts (HELF) [33], while PC mediates HCMV entry into epithelial/endothelial cells [35]. Recently, both TC and PC have been reported to promote a two-step process for HCMV entry into both epithelial and endothelial cells [36].

Following the identification of the two gCIIIs (gHgLgO and gHgLpUL128L), a study was undertaken to investigate the ELISA IgG reactivity of sequential sera from pregnant women, within the first year after onset of HCMV PI, to soluble forms of PC and gB as well as neutralizing activity preventing the infection of both epithelial/endothelial and HELF cells [37,38,39]. Results showed that ELISA IgG antibodies to gB increased more rapidly and at higher titers compared to antibodies to PC, while NAb preventing the infection of epithelial/endothelial cells appeared much earlier and at higher titers than NAb preventing the infection of HELF [27]. Following the documentation of a fair degree of correlation between anti-PC ELISA-IgG antibodies and NAb preventing the infection of epithelial/endothelial cells, a series of preabsorption experiments with PC in convalescent-phase sera from primary infections showed that >90% of the neutralizing activity was due to PC, whereas it was not affected by preabsorption with gB [27].

In addition, when a group of NT women was compared to a group of T women, it was found that IgG Ab titers to PC were significantly higher in NT compared to T pregnant women in the first 30 days after onset of infection, whereas IgG antibody titers were not different in the two groups, and the viral load was significantly higher in T compared to NT women. Vertical transmission to the fetus was further analyzed by using the competitive ELISA inhibition of monoclonal antibody binding (IMAB) assay. In this test, the PC is bound to the solid phase and reacted competitively with human sera and murinized human monoclonal antibodies [39]. Using this assay, it was shown that all HCMV seropositive individuals are reactive with all 10 neutralization antigenic sites identified on PC by a panel of neutralizing mAb. IMAB titers in the two groups of T and NT pregnant women examined were found to be significantly lower in the T group for 7/10 sites, while the number of neutralization sites recognized by T women was significantly lower compared to NT women during the first and the second month after onset of infection [27]. Furthermore, the inhibiting activity of both human mAbs and convalescent-phase sera from HCMV PIs was studied in the two groups of T and NT pregnant women with the aim of evaluating the control of virus dissemination occurring through the following mechanisms: plaque-formation inhibition (PFI), leukocyte transfer inhibition (LTI) [37], and syncytium formation inhibition (SFI) [38]. It was found that LTI antibodies [39] and SFI antibodies [38] appeared late, i.e., at least 30 days after onset of PI, whereas PFI antibodies were detectable much earlier, and at significantly higher titer in NT compared to T women during the first month after infection onset [27]. This inhibitory effect was also displayed by human mAbs directed to PC, but not by mAbs anti-gB. These results document that neutralizing antibodies may limit virus dissemination through different inhibitory mechanisms of cell-to-cell spreading (PFI), HCMV transfer to leukocytes from infected endothelial cells of the vascular tree (LTI) and syncytium formation (SFI).

### 4.2. Cellular Immunity

Cellular immune response to HCMV PI includes both innate and adaptive T-cell responses. Natural killer (NK) cells are the innate immune cells mostly interested in the immune response during HCMV PI. In particular, a stable expansion of NK cells NKG2C^bright^ CD57^+^ has been reported early in life as well as in congenitally infected children [40,41]. In our unpublished study, it was observed that the absolute number and the percentage of NKG2C^bright^ CD57^+^ cells were significantly higher in seropositive subjects compared to seronegative ones [42]. NK cells are considered the main effectors of antibody-dependent cell-mediated cytotoxicity [43].

In addition, γ/δ T cells, and in particular Vδ2^−^ (and not Vδ2^+^) γ/δ T cells, which share some properties with both innate and adaptive T cells, have been shown to undergo a long-lasting expansion upon HCMV PI in both immune-competent and immune-compromised patients [44] as well as in pregnant women [45] and congenital infections [46]. Their role in HCMV transmission to the fetus remains to be defined.

The adaptive T-cell immune response to primary HCMV infection has been investigated by our group by either evaluating the lymphoproliferative response (LPR) to HCMV-infected cell lysate or by using a method developed in-house, the infected dendritic cell method [47]. This method, based on the incubation of patient PBMC with autologous monocyte-derived, HCMV-infected dendritic cells, was followed by determination of multiple membrane/intracellular markers by cytokine flow cytometry using fluorochrome-conjugated mAbs. Our studies, though not able to document a condition of immune depression during pregnancy [48], reported a significant association between a delayed LPR and HCMV transmission to the fetus (Table 1(1)) [48,49].

Furthermore, it is well known that different T-cell subpopulations can be identified through the analysis of the surface expression of chemokine receptor CCR7 and the different isoforms of CD45 [50]. Naïve T cells CCR7^+^ CD45RA^+^ may migrate from CD45RA isoform to CD45RO, upon their first contact with the antigen, while memory T cells, according to the different expression of CCR7, may be divided into: central memory T cells (T_CM_ CD45RA^−^CCR7^+^) and effector memory T cells (T_EM_ CD45RA^−^CCR7^−^). T_CM_ cells display a high proliferation potential and can migrate to lymph nodes, while T_EM_ cells can revert to the RA isoform of CD45 after new contact with the antigen (T_EMRA_ CD45RA^+^ CCR7^−^) [51]. While in a first study we detected a higher percentage of HCMV-specific CD4^+^ T_EMRA_ T cells in NT pregnant women [52], in a second study conducted on a greater number of pregnant women, the percentages of both HCMV-specific IFN-γ^+^ CD4^+^ and CD8^+^ CD45^+^ T_EMRA_ T cells were significantly higher in NT women (Table 1(3,4)). Furthermore, in NT women at 30 d after infection onset there was a significantly higher IL-2 production by HCMV-specific CD4^+^ T cells (Table 1(2)) [53].

**Table 1:** Immune correlates of protection against HCMV transmission to the fetus in groups of non-transmitting (NT) and transmitting (T) pregnant women with PI.

Parameters (Days after Infection Onset ^a^)	NT			T			*p*
	N ^c^	Median (Range)	Average ± SD	N ^c^	Median (Range)	Average ± SD	
1. LPR CD4^+^ CDI ^b^ (30–90) [49]	21	6 (0–47)	13 ± 13	16	3 (0.02–23)	6 ± 7	<0.05
2. IL-2^+^ CD4^+^ % (30) [53]	27	20 (0–60)	24 ± 19	11	11 (0–40)	11 ± 12	0.05
3. CD45RA^+^ CD4^+^ % (60) [53]	48	19 (3–95)	15 ± 16	26	13 (0–36)	15 ± 11	<0.05
4. CD45RA^+^ CD8^+^ % (60) [53]	48	49 (10–98)	52 ± 21	26	34 (11–73)	38 ± 18	<0.01
5. IL7R^+^ CD4^+^ % (30) [54]	10	72 (26–94)	67 ± 21	10	37 (7–58)	35 ±14	<0.01
6. no. HCMV pp65-specific spots/10^6^ PBMC ^d^ (30–60) [55]	29	388 (0–8867)	861 ± 1700	15	13 (0–1150)	180 ± 308	<0.01

**^a^** time significantly different between T and NT women; **^b^** cell division index; **^c^** number of pregnant women; **^d^** cultured ELISPOT assay.

In conclusion, according to results of our studies, the delayed expression of at least three immunologic markers, such as LPR, IL-2 production by HCMV-specific CD4^+^ T cells and the re-expression of CD45RA in HCMV-specific (IFN-γ^+^) CD4^+^ and CD8^+^ T cells, appears to be associated with HCMV vertical transmission. However, at this time, none of these immune correlates of protection can be taken into consideration on an individual basis for prognostic purposes, due to the variation in individual values of these parameters in pregnant women [42]. A more extended evaluation of the prognostic potential of these and other parameters would better define the limits of these conclusions.

Finally, other approaches were investigated for detecting new immune correlates of protection against HCMV transmission to the fetus. In particular, while the study of the T cell response to 4 major structural and nonstructural proteins using peptide pools did not show any association between T cell antigen specificity and vertical transmission, a comparative study of the two IL-7R subsets (IL-7R^pos^ and IL-7R^neg^) of CD4^+^ and CD8^+^ T cells showed a significantly higher percentage of IL-7R^neg^ HCMV-specific CD4^+^ T cells in T women, whereas no difference was reported for the percentage of IL-7R^neg^ HCMV-specific CD8^+^ T cells between T and NT mothers. Thus, it was concluded that a lower percentage of IL-7R^neg^ CD4^+^ T cells and a higher percentage of IL-7R^pos^ CD4^+^ T cells are the result of a better control of virus infection in NT women (Table 1(5)) [54].

## 5. Diagnosis

### 5.1. Diagnosis of Maternal Infection

As mentioned above, cCMV may be the result of either a maternal PI in pre-pregnancy seronegative mothers or NPI in pre-pregnancy seropositive mothers. While in PI the rate of HCMV fetal transmission ranges at around 30–40% [8,9,56], in NPI the transmission rate is about 1% [57] or <4% [58]. Thus, how is the rate of cCMV due to NPI comparable and even superior to that due to PI? [26]. The reason for this discrepancy may reside in the fact that, while PI does occur only once in a lifetime, reactivation/reinfection episodes may be multiple. The same explanation may hold true for the same rate of neurodevelopmental *sequelae* and SNHL in PI and NPI. However, several recent papers have reported that an estimation of the placental transmission rate in NPI is lower than that of PI [58,59].

In order to differentiate between PI and NPI during pregnancy, it would be mandatory to know the HCMV serologic status of the women prior to pregnancy. A seronegative woman would be at risk for PI during pregnancy with a high rate of virus transmission to the fetus, whereas a seropositive woman would have a much lower risk of fetal transmission. If serologic testing is performed during pregnancy, it must be done early in pregnancy, i.e., within 12–14 weeks of gestation [60,61].

**Figure 1:**
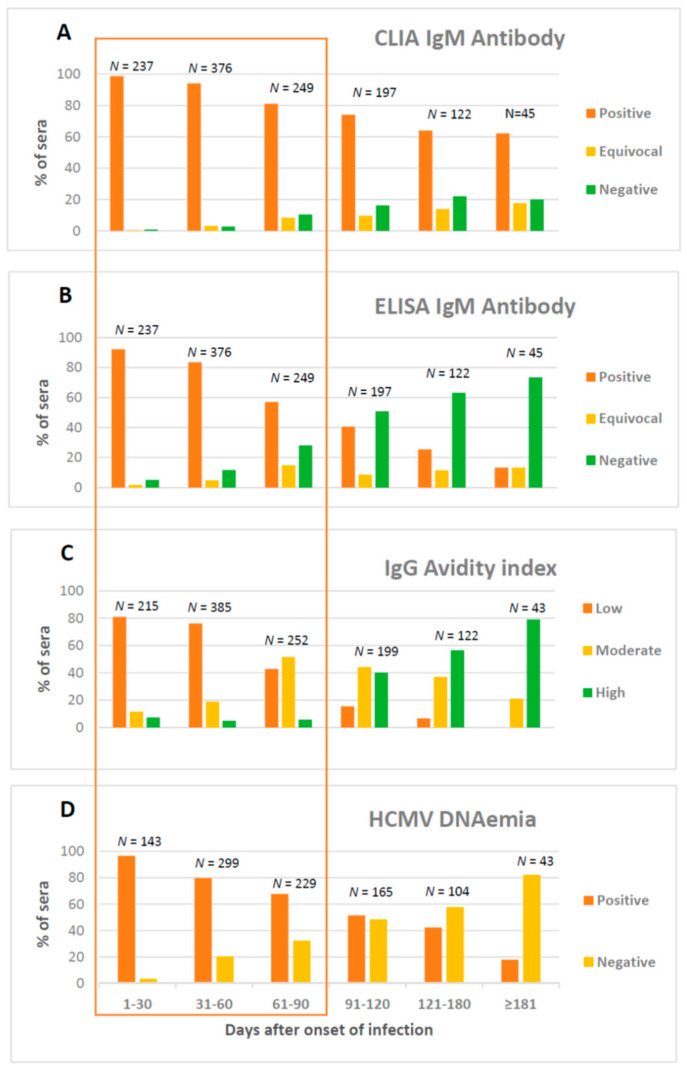
(**A**) HCMV chemiluminescent immunoassay (CLIA) for IgM antibody; (**B**) enzyme-linked immunosorbent assay (ELISA) for IgM antibody; (**C**) IgG avidity index; and (**D**) DNAemia. Results as obtained on sequential serum and whole blood samples from 465 pregnant women with HCMV PI [63].

Serologic diagnosis of PI is based on three major endpoints: IgG seroconversion, presence of HCMV-specific IgM antibody and low AI (Figure 1).

*IgG Seroconversion.* IgG seroconversion requires sequential serum samples, which are often difficult to obtain. In addition, in a recent report, a false IgG-seropositivity at prenatal screening, using a highly automated commercial assay, was detected in 11/678 (1.6%) women who tested negative with confirmatory assays [62]. Thus, some warnings should be raised about IgG testing: (i) HCMV-specific IgG seroconversion in the absence of other diagnostic parameters (HCMV-specific IgM and/or virus DNA) is not *per se* suggestive of PI, but requires further testing; (ii) laboratory results should include technical characteristics of the methodology used; (iii) low levels of specific IgG in the absence of IgM antibodies should be further investigated with additional assays, such as immunoblotting and NAb assay; (iv) in the absence of an international IgG standard, results among different laboratories are not comparable; (v) should one of these women with false IgG seropositivity deliver a newborn with cCMV, the congenital infection would be considered the result of an NPI.

*IgM antibody.* IgM antibody detection as a single reference parameter has several limitations due to the fact that IgM, besides sometimes producing false-positive results, during the first trimester of pregnancy may have too short or too long a detection window and may also occur during episodes of HCMV reactivation/reinfection. In clinical practice, detection of HCMV-specific IgM antibodies, as a rule, requires an interpretation, because it could be due not only to a PI, but also to: (i) NPI or persistence of IgM after PI; (ii) polyclonal activation or cross-reactivity during other infections, such as rubella, parvovirus B19, or others. Thus, serum testing with another test format is requested [63] (Figure 1A,B).

*AI*. Low AI during the first trimester of pregnancy will detect most pregnant women with PI who are at risk of delivering an infected newborn with cCMV and SNHL [2]. However, in a very recent study on 465 pregnant women with PI, 20/426 (4.7%) and 26/418 (6.2%) showed undetectable IgM antibody levels and high IgG AI, respectively, following testing within 1–3 months after careful determination of infection onset [63]. Therefore, almost 11% of the pregnant women showed misleading serological results, i.e., early IgM antibody clearance and early IgG AI maturation (Figure 1C). The lack of an international standard serum panel and the variability of results among laboratories using different commercial assays [57,64] often generated challenging AI results, which should be considered contextually with the other assays (HCMV-specificIgG and IgM antibodies) in view of making a reliable diagnosis of PI. In conclusion, some major points on the use of AI in clinical practice must be considered. Firstly, it is important not to perform the AI assay in serum containing low levels of IgG antibodies. Secondly, even though several studies reported a good concordance among different commercial avidity assays [65,66,67], our findings suggest that, often, different results are obtained when using different AI assays. Thirdly, to avoid the risk of missing a case of PI, we recommend to test both for IgG- and IgM-specific antibodies, and, in case of good IgG reactivity (>than 3× the cutoff value), complete the screening with the AI, regardless of the presence of IgM antibody.

*NAb.* Finally, an additional serological parameter which could help in the diagnosis of PI is the absence of neutralizing antibodies (NAb), as determined in HELF cells, in maternal blood for 30–60 d after onset of PI [37,68].

*DNAemia.* Although prolonged maternal DNAemia in PI has been reported to be associated with risk of vertical transmission, quantification of HCMV DNAemia by qPCR in maternal blood can only play a role of support to serological diagnosis [69,70,71]. However, according to our experience, presence of HCMV DNAemia should always elicit the suspicion of PI (Figure 1D) [56,63,72]. In addition, currently, the role of viral load quantification in blood is not defined in the context of diagnosis of NPI in pregnant women who are seropositive prior to pregnancy.

*DNA in bodily fluids.* As for detection of HCMV DNA in bodily fluids, 15 pregnant women with PI were sequentially tested and found to harbor HCMV DNA in saliva, urine or genital swab until 12 months after onset of PI. On the other hand, when virus shedding in bodily fluids was studied in a group of seropositive pregnant women who were IgM-negative with a high AI at the beginning of pregnancy, about 30% were DNA-positive in bodily fluids during all three trimesters of pregnancy, thus suggesting a recurrent (non-primary) infection (unpublished results). Due to the presence of HCMV DNA either in PI or NPI, searching for viral DNA in bodily fluids should not be routinely performed in clinical practice.

**Figure 2:**
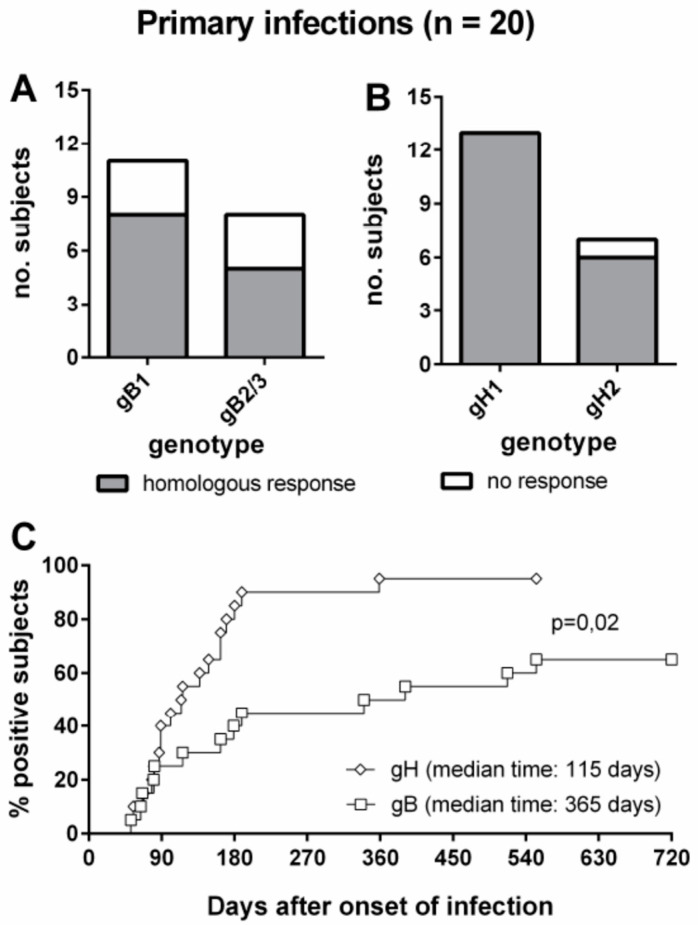
Genotype-specific IgG antibody to gB and gH in subjects with HCMV PI. (**A**): gB genotype-specific IgG response in subjects infected by gB1 and gB2/3 HCMV strains. (**B**): gH genotype-specific IgG response in subjects infected by gH1 and gH2 HCMV strains. (**C**): Comparative times to development of genotype-specific IgG responses to gB and gH [74].

*NPI diagnosis.* Current serological techniques do not permit diagnosis of NPI due to HCMV reactivation/reinfection episodes. However, the detection of IgG antibodies with specific reactivity to some epitopes of gH and gB has been proposed as a new serological tool for diagnosis of NPI [73]. On this basis, recently, using a peptide-based ELISA, detection of genotype-specific antibodies to gB and gH was reported to be suitable for identifying NPI, although about half the subjects did not have genotype-specific IgG antibodies to gB (Figure 2A–C) [74].

*Timing of PI.* At present, once diagnosis of PI is established, it appears critical to define the onset of PI for both prognostic and pregnancy management reasons. To this aim, both anamnestic findings and clinical history, along with serological and virological data, may help in defining the gestation timing at onset of PI. Even though PI is commonly asymptomatic, Daiminger et al. [75] concluded that in the first part of pregnancy, symptoms of any kind should suggest the appropriateness of a serological screening for HCMV. In our experience, non-specific symptoms (fever, headache, asthenia and upper respiratory symptoms) as well as alterations of biochemical/hematological parameters (hypertransaminasemia, leukocytosis and thrombocytopenia) were found in 530/721 (73.5%) pregnant women with PI in one study [69] and in 72/163 (58.5%) in another study [56]. Thus, when a PI is suspected, both anamnesis and abnormal laboratory findings should be carefully evaluated, not only to diagnose a PI, but also to define its timing (onset). In this case, what is important is to test pregnant women no later than at 12 weeks of gestation and possibly as early as possible in order to define whether PI occurred prior to or after pregnancy initiation [76]. In an old study conducted on the diagnosis and outcome of preconceptional and periconceptional HCMV PI, it was reported that only 1/11 (9.1%) newborns of women with preconceptional PI had been congenitally infected, whereas 4/13 (30.8%) newborns of women with periconceptional PI had cCMV. The conclusion of that study was that periconceptional PI seemed to bear a higher risk of HCMV vertical transmission than preconceptional PI [76].

Timing of PI is crucial for assessing the risk of vertical transmission. In our experience, by combining serologic, virologic (viral DNAemia), anamnestic and laboratory findings, we were able to date the onset of PI in 695/735 (94.6%) women [69]. For Daiminger et al. [75], the timing of PI was based on the following: (i) seroconversion of HCMV-specific IgG and its kinetics; (ii) kinetics of HCMV-specific IgM; (iii) absence or increasing levels of NAb; (iv) low IgG AI or increasing levels of IgG AI. More recently, Delforge et al. [67] determined onset of PI in 40 pregnant women based on the following criteria: (i) IgG seroconversion, or when both IgG and IgM are detected at first positive serology. The onset of PI was set at the mid time between the last IgG -negative and the first IgG-positive serum sample; (ii) when only IgM antibodies were detected at first positive serology, onset of PI was set at that point; (iii) in the presence of clinical symptoms and/or biochemical alterations compatible with HCMV infection as well as specific IgM antibodies and a significant rise in IgG, the onset was set at the time of symptoms.

**Figure 3:**
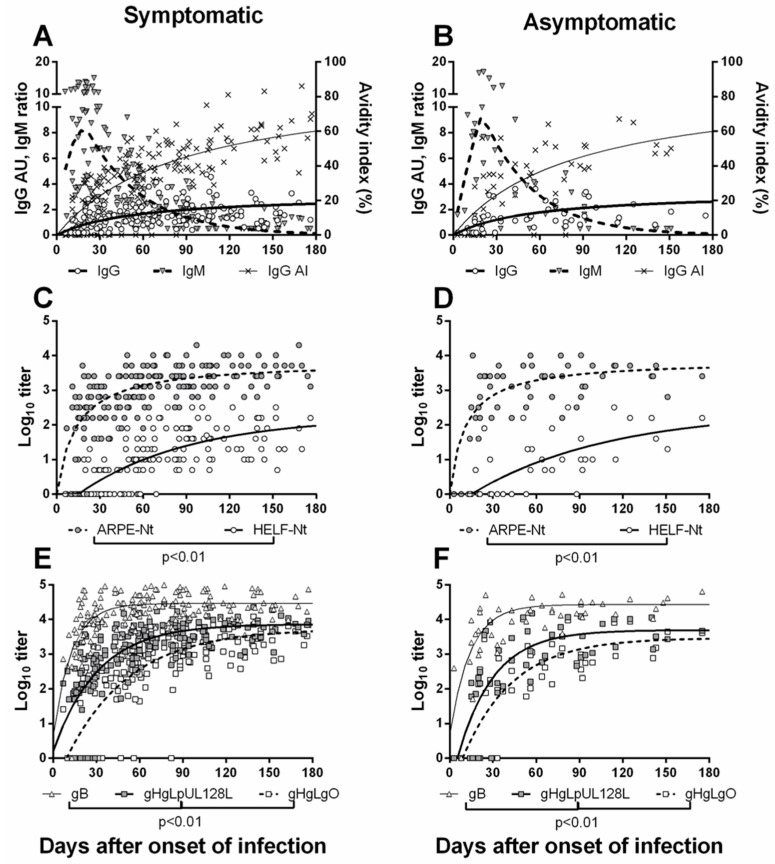
Antibody kinetics according to different serological assays during the first 180 days after PI onset in 40 pregnant women with symptomatic (left panel) and 13 pregnant women with asymptomatic (right panel) infection. (**A**,**B**): HCMV-specific IgG and IgM antibodies and IgG avidity index (AI). (**C**,**D**): Neutralizing (Nt) antibody titer, as determined in ARPE-19 epithelial cells and HELF cells. (**E**,**F**): ELISA IgG antibody titer to VR1814 glycoprotein complexes gB, gH/gL/pUL128L and gH/gL/gO [77].

Our group evaluated the differential kinetics of additional parameters (NAb in HELF and ARPE-19 cells and ELISA-IgG antibodies to TC, PC, and gB) with the aim of establishing criteria for dating PI onset in pregnant women in the absence of clinical symptoms (Figure 3) [77]. Results showed that, while antibodies to gB and PC as well as NAb preventing infection of ARPE-19 epithelial cells appeared early (within 2–3 weeks) after infection onset and increased rapidly, antibodies to TC and NAb preventing infection of HELF appeared late (>30 d) and increased slowly. As a result, the delayed appearance of NAb preventing infection of HELF in combination with the other diagnostic parameters (IgG and IgM antibodies, AI, DNAemia and careful anamnesis) has been used as an additional assay to detect PI onset occurring <6 wks in advance [63]. On the other hand, analysis of the T-cell response, and in particular of certain delayed phenotypical and functional features, during the early pregnancy phase, may also help in dating PI [53]. In this respect, while serological parameters allow diagnosis of PI (when serum samples are obtained within 3 months after infection onset), dating of PI is much more difficult in the case of delayed diagnosis during pregnancy (after 3–6 months or more). In a search for immune parameters able to discriminate between primary and remote infections, it was reported that at 180 d after infection onset, CD4^+^ lymphoproliferation and IL-2 producing HCMV-specific CD8^+^ T cells were the best parameters for discriminating between PI and remote infection [53].

**Figure 4:**
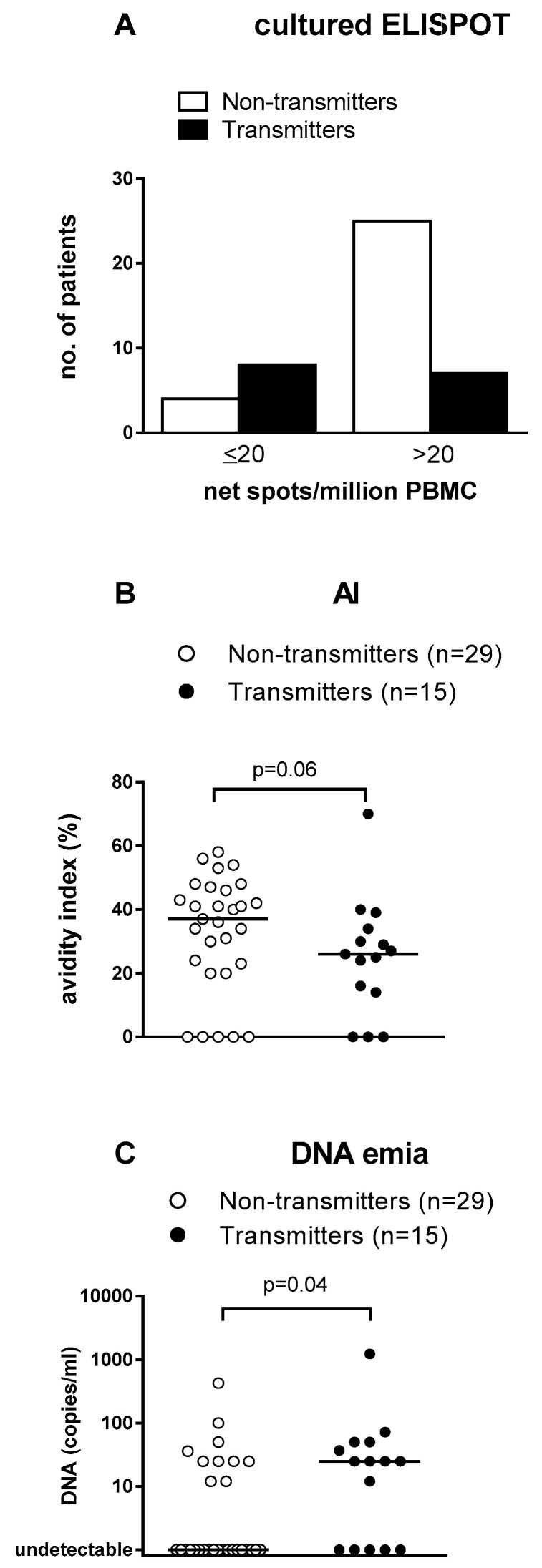
(**A**): Cultured ELISPOT assay. Using a pp65-specific T cell determination, it was found that pregnant women with >20 spots/10^6^ PBMC transmitted the infection to the fetus at a significantly lower rate than women with <20 spots/10^6^ PBMC. (**B**): among serological parameters, IgG AI was higher in NT compared to T women. (**C**): viral load in blood was significantly lower in NT compared to T women [55].

*Prognostic parameters of cCMV.* In the search of maternal parameters prognostic of virus transmission, recently [78] Penka et al. [78] evaluated the placenta-derived growth factor (PIGF) and soluble fms-like tyrosine kinase 1 (sFlt1) concentrations in the maternal serum of 114 women with PI, finally suggesting that an invasive prenatal diagnosis with amniocentesis should be performed in the presence of high levels of these parameters. In one retrospective study conducted on 150 newborns of whom 28 (18.7%) were symptomatic at birth, it was shown that the risk of symptomatic cCMV at birth increased linearly with the number of significant maternal (gestational age at infection), fetal (viral DNA levels in blood and amniotic fluid, and IgM antibody level) and neonatal (DNAemia) parameters [79]. A similar result was reported by Simonazzi et al. [70] who showed that maternal DNAemia in a group of 239 pregnant women with PI is associated with a 3-fold greater chance of cCMV infection. In another study, determination of HCMV-specific T cells by cultured ELISPOT (response to pp65, but not to IE-1 or IE-2) was significantly higher in NT mothers (Table 1(6)), while among different serologic parameters examined, only IgG AI was higher in NT mothers, who also showed a lower DNAemia level (Figure 4) [55]. In a multivariate logistic regression analysis, the three parameters (highly cultured ELISPOT response to pp65, high AI, and low DNAemia) were independently associated with lack of vertical transmission.

### 5.2. Prenatal Diagnosis of cCMV

Prenatal diagnosis of cCMV is aimed at verifying the HCMV transmission to the fetus by using either invasive and/or noninvasive approaches. Invasive prenatal diagnosis includes *amniocentesis* and/or *cordocentesis*, while noninvasive prenatal diagnosis refers to *ultrasound imaging*.

*Amniocentesis* is the procedure of election for diagnosis of fetal infection by detection and quantification of viral DNA by qPCR in amniotic fluid (AF) in an interval between 8 weeks after onset of maternal primary infection and 20–21 weeks of gestation [69,80,81]. However, even when the invasive procedure is performed at the indicated gestation time, a small number of false-negative prenatal diagnosis results cannot be avoided. In our experience, about 8% of negative prenatal diagnosis results are not confirmed at birth, even though the neonatal outcome is favorable, with no long-term *sequelae* [82].

*Cordocentesis* may represent a second invasive approach to the diagnosis of cCMV; however, it is not recommended due to the risk for the fetus. However, when performed, since ultrasound (US) findings are normal in most cases of fetal infection at prenatal diagnosis [83], additional biochemical, hematologic and virologic testing may provide useful prognostic markers for differentiating symptomatic from asymptomatic cCMV. The best nonviral markers were β2-microglobulin (above 11.5 mg/L) and platelet count (below 50.000/μL), while the best virologic markers were a high level of IgM antibody index (by ELISA) and DNAemia (above 30,000 copies/mL) [84]. Obviously, these prognostic markers should be determined in fetal blood after virus detection in AF by amniocentesis, in view of predicting the perinatal outcome of cCMV.

*Ultrasound imaging.* Noninvasive prenatal diagnosis is based on US imaging, which can help in the diagnosis of cCMV in about 15% of the cases [85] by detecting placentitis, oligohydramnios, hepatosplenomegaly and other minor findings. US examination in cCMV infection can help in monitoring fetal abnormalities [86], but also in predicting normal neurodevelopmental outcomes [87]. However, only the combined use of serial US and magnetic resonance examinations provides 95% sensitivity in detecting CNS abnormalities after 30 weeks of gestation [88]. In a recent study on blood samples collected from 34 congenitally infected fetuses, it was reported that high levels of β2-microglobulin and low platelet counts were significantly more often found in fetuses with severe US abnormalities compared with fetuses with no or mild abnormalities. In addition, symptomatic infected fetuses had higher IgM index values and higher viral load [81].

As already reported above, the immune correlates of protection from virus transmission to the fetus during maternal PI in pregnancy, while significant on a statistical basis, cannot be referred to on an individual basis [42].

### 5.3. Diagnosis of cCMV in the Newborn Infant

cCMV is diagnosed in the newborn infant either for confirmation of prenatal diagnosis or detection of virus transmission to the fetus during PI or NPI of the mother during pregnancy. The gold standard diagnostic assay was initially virus recovery/isolation in HELF cell cultures following inoculation of clinical urine samples collected within 2 weeks of life either by conventional procedures [89] or rapid techniques [90,91,92]. In the meantime, saliva was reported to be a much more easily available sample than urine for diagnosis of cCMV [93]. In addition, at the end of the 1980s, PCR for HCMV DNA detection in urine was shown to be 100% sensitive and specific when compared to conventional virus isolation procedures [94]. Furthermore, saliva was reported in a large study to be as sensitive as urine for HCMV DNA detection [95,96,97,98].

These results opened the way to screening programs for detection of cCMV in asymptomatic newborns. There is still some debate on the opportunity of implementing targeted screening, such as for SNHL, or universal screening. The second option has been reported to be more cost-effective. A third option is testing of dried blood spots (DBS) that are routinely collected from all newborns for metabolic screening. A series of retrospective studies indicated that detection of viral DNA in DBS could be used for diagnosis of cCMV in newborns [99,100,101]. However, methods of viral DNA extraction from DBS have been shown to affect the analytic sensitivity of the assay [102]. In addition, in an extended study performed on more than twenty thousand newborns, DBS PCR was less sensitive than saliva rapid cell culture [101].

## 6. Prevention

At this time, four substantially different approaches to the major objective of preventing cCMV have been identified: (i) educational and hygienic measures; (ii) use of hyperimmune human immunoglobulin (HIG) preparations; (iii) use of an antiviral therapy, consisting of oral administration of an anti-herpes drug, valaciclovir; (iv) vaccines.

*Educational and hygienic measures.* In the past decades, the issue of prevention of HCMV PI by educational interventions was addressed in very few studies. In the USA, two small studies were conducted by SP Adler et al. in the 1990s, when a reduction in the rate of PI was reported in pregnant women following hygiene recommendations compared to non-pregnant women [103,104]. A subsequent French study documented a higher rate of PI at 12 weeks gestation in a group of pregnant women without hygiene education (0.42% in the interval 0–12 weeks gestation) in comparison with the rate of PI between 12 and 36 weeks of gestation in another group of pregnant women following hygiene counseling (0.19% in the interval 13–36 weeks gestation) [105]. Unlike these studies, where both educational interventions and study populations were different, more recently, we performed a study in which the interventional arm included HCMV-seronegative women who were enrolled at the time of maternal serum screening for fetal aneuploidy at 11–12 weeks of gestation, while the observational comparison arm included women enrolled at delivery who were not informed about HCMV, but had a serum sample stored at the screening for fetal aneuploidy. Both groups were homogeneous for age, parity, education and exposure to risk factors. In detail, in the intervention arm participants were invited to frequently wash their hands after exposure to young children’s bodily fluids as well as surfaces touched by children (toys, high chair, stroller, etc.). Women were also invited to avoid kissing children on the mouth/cheeks and not to share utensils, food, drinks, washcloths etc. Results showed that 4/331 (1.2%) seroconverted in the interventional group compared to 24/315 (7.6%) in the comparison group (*p* < 0.01), thus providing evidence that the intervention based on hygiene counseling significantly prevented maternal infection [106]. The adoption of the above-mentioned hygienic measures aimed at limiting the contacts with bodily fluids of young children and implementing a frequent hand-cleaning behavior after children care is simple, well-tolerated by pregnant women and highly effective.

*HIG preparations.* The initial report by Nigro et al. [107] on two groups of pregnant women with maternal PI diagnosed at <21 weeks of gestation (a control group and an intervention group) showed that, using a four-weekly HIG regimen for prevention of HCMV vertical transmission in the intervention group, 6/37 (16%) women receiving HIG had infants with cCMV, compared to 19/47 (40%) who did not receive HIG. Thus, HIG administration was associated with a significantly lower risk of cCMV (*p* = 0.04). Similarly, 1/31 (3.2%) pregnancies receiving HIG resulted in symptomatic cCMV in infancy, whereas 7/14 (50.0%) pregnancies not receiving HIG resulted in symptomatic cCMV in infancy. Subsequently, other early observational studies reported encouraging results using HIG [108,109,110]. However, a randomized double-blind study conducted by Revello et al. [56] showed a non-significant reduction in cCMV in pregnant women receiving HIG compared to the group not receiving HIG (30% vs. 44%; *p* = 0.13). In addition, a more extended phase III placebo-controlled double-blind study was undertaken to evaluate the role of HIG in preventing fetal HCMV infection in pregnant women with PI acquired prior to 24 weeks of gestation (clinicaltrials.gov: NCT01376778, accessed on 29 May 2021). However, this trial was prematurely stopped, since it was predicted that no significant difference was likely to be detected for cCMV in pregnant women receiving HIG compared to pregnant women receiving placebo (22.7% vs. 19.4%, respectively; *p* = 0.42) [111].

In both of the above mentioned RCTs failing to demonstrate a protective effect of HIG administration in reducing the vertical transmission rate, the study protocol was similar, including an HIG dosage of 100 IU/kg bodyweight administered on a monthly basis up to the end of pregnancy. Based on the new pharmacological study indicating that the half time of HIG is only about 10 days [112,113], a new investigation was recently undertaken in 149 pregnant women and 153 fetuses to study the efficacy of HIG in pregnant women with a very recent PI in the first trimester or during the periconceptional period with treatment starting at a median gestational age of 10.6 weeks and ending at 17.9 weeks. During this time, IV treatment with HIG was administered on the average only 4 times (every 2 weeks) until about 18 weeks of gestation at the HIG dosage of 200 IU/kg bodyweight [114]. Very recent PI in the first trimester of pregnancy along with timely initiation of treatment and an appropriate treatment dosage and interval were the parameters preventing maternal-fetal transmission. Results of this study preliminarily indicated that vertical transmission occurred only in 10/153 fetuses enrolled (6.5%).

*Antiviral drugs: valaciclovir.* Notwithstanding the elevated number of antiviral agents available for treatment of HCMV infections/disease in non-pregnancy settings, very few data are available for justifying their use in pregnancy [115]. The only agent thus far showing good oral bioavailability [116] and no association with birth defects in population studies is valaciclovir [117]. Between 2015 and 2018, a randomized double-blind placebo-controlled trial was performed to investigate whether valaciclovir can prevent vertical transmission of HCMV to the fetus in pregnant women with PI periconceptional or acquired in the first trimester of pregnancy (clinicaltrials.gov: NCT02351102, accessed on 29 May 2021) [118]. Final analysis included 45 patients enrolled in the valaciclovir group (8 g/day, twice daily) and 45 patients in the placebo group from enrollment until amniocentesis at 21–22 gestational weeks. In the valaciclovir group 4/45 (11%) amniocenteses were HCMV-positive compared to 14/47 (30%) amniocenteses which were positive in the placebo group (*p* = 0.027; OR 0.29). It was concluded that valaciclovir is effective in reducing HCMV transmission to the fetus following maternal PI acquired early in pregnancy. On this basis, the Italian government decided on 20 December, 2020 to include valaciclovir at its own expense in the list of drugs for the in utero prevention and treatment of cCMV [119]. On the other hand, a study by Roxby et al. [120] on HIV-infected women did not report a significant reduction at birth in HCMV transmission to infants in pregnant women receiving valaciclovir prophylaxis compared to the placebo controls.

*Vaccines.* For several decades, the development of an effective HCMV vaccine able to prevent PI as well as reactivations and reinfections both in developed and developing countries has been a major priority for public health authorities to achieve in order to decrease rates of cCMV, as well as rates of HCMV disease in the transplantation setting. While for most viruses, such as rubella, measles and mumps, the natural infection provided lifelong protection and vaccination aimed at providing the same level of immunity, in the case of HCMV and the other human herpesviruses, the virus strain originally infecting an individual is not eliminated from the body following PI resolution, but persists in a latency state (as DNA), periodically restarting its replication cycle with final release of mature virions. This implies occurrence of repeated episodes of recurrence or reactivation. However, PI does not confer protection either against reinfection by new virus strains, often concomitantly with factors debilitating the immune system. As a result, until we fully understand the pathogenetic basis of the partial protection given both by natural infection and vaccination, we will not reach sterilizing immunity. On the other hand, the immune response to HCMV infection has been shown to involve both humoral and cellular immunity (see above).

Along the years, several different approaches to the development of HCMV vaccines have been undertaken. Basically, two groups of vaccines were developed: living and non-living vaccines. Among the live HCMV vaccines, we must list: (i) the *Towne vaccine* developed by Plotkin [121] and showing partial protection; (ii) the *AD-169 (V160*) *vaccine* developed by Elek&Stern [122], restored in its mutated PC [123] and modified by incorporating a synthetic compound conditioning its replication (*V160*); (iii) *Towne/Toledo chimera vaccines* constructed by replacing genomic regions of the wild-type Toledo strain with attenuated Towne strain genomic regions [124]; (iv) *viral vectored HCMV vaccines*, in which the heterologous vector may be canarypox, alphavirus Venezuelan equine encephalitis (VEE), lymphocyte choriomeningitis (LCM) virus, modified vaccinia Ankara (MVA) virus, adenovirus type 6; and (v) *alphavirus replicon particles (VRPs) vaccine*, which is an RNA-based vaccine consisting of an attenuated strain of the VEE virus, in which structural VEE protein genes are replaced by genes encoding for HCMV proteins.

Within the group of *live HCMV vaccines*, the vaccine in an advanced clinical development stage is V160. This vaccine, derived from the AD-169 live attenuated virus, was restored in its PC, while a molecular switch was introduced into two HCMV genes, IE-1 and UL151 [125], allowing complete virus replication during vaccine production, but blocking virus replication following vaccine inoculation [126]. After administration of three doses of V160 vaccine, in a Phase II clinical trial, a good NAb and strong and persisting HCMV-specific IE-1 and pp65 T-cell responses (predominant CD8^+^ T cells with effector phenotypes) were observed [126].

The other group of vaccines (non-living vaccines) included: (i) *gB subunit vaccines* first developed at Chiron and then modified at Novartis and Pasteur; (ii) *DNA-based vaccines* including a series of plasmid-based vaccines; (iii) *RNA-based vaccines* formulated with lipid nanoparticles; (iv) *virus-like particle (VLP) vaccines* consisting of enveloped virus-like particles simulating wild-type viruses, but lacking viral genome; (v) *dense body vaccines* consisting of enveloped dense bodies present abundantly in HCMV-infected cells at a late stage of infection; (vi) *peptide vaccines* mostly directed to protection from HCMV disease in HSCT recipients; and (vii) *PC vaccines*, based on the property of PC to be the major HCMV neutralization antigen, which was required for infection of both endothelial and epithelial cells [34,35].

Among *non-living HCMV vaccines*, one of the most widely studied has been the recombinant gB subunit vaccine. First developed by Chiron using gB from the Towne strain, it was administered in the oil-in-water adjuvant MF59 to both adults and toddlers, but the antibody response was short-lived and waned within a year [127]. Subsequently, Novartis and then Sanofi Pasteur modified the gB subunit vaccine by removing the transmembrane domain and the furin cleavage and fusing the cytoplasmic component of the transmembrane domain with the truncated gB. This modified gB was then used in several Phase II clinical studies. In one study, 50% protection against HCMV PI was shown in postpartum seronegative women, along with a reduction in cCMV [29]. In addition, in the same study, after vaccination, seropositive women showed a boosted gB-specific immune (both humoral and cellular) response [128]. Further, when administered to seronegative adolescent girls, the gB/MF59 vaccine induced a reduction, although not significant, of PI compared to the control group [129]. A similar benefit was observed also in SOT recipients [30].

Within *RNA-based vaccines*, a synthetic self-amplifying *HCMV mRNA vaccine* expressing gB and a pp65-IE-1 fusion construct was first developed by Novartis (now GSK). After two IM immunizations in rhesus macaques, all animals showed good immune responses both humoral (IgG and NAb) and cellular (both CD4^+^ and CD8^+^ T cells). Another mRNA-based HCMV vaccine platform using self-amplifying mRNA was developed by Moderna Therapeutics. In this vaccine, mRNA was formulated with lipid nanoparticles encapsulating mRNAs encoding gB and PC. Immunization of non-human primates induced durable NAb response [130] (clinicaltrials.gov: NCT03382405 and NCT04232280, accessed on 29 May 2021). The administration of an additional mRNA vaccine expressing the HCMV immunodominant T cell antigen pp65 together with the mRNA vaccine expressing gB and PC induced both a potent humoral and cell-mediated immune response in mice [130].

Variation Biotechnologies Vaccines Incorporated (VBI) developed some *eVLPs* vaccines. A gB vaccine was developed using eVLP by co-transfecting HEK-293 cells with a plasmid encoding murine leukemia virus Gag (generating VLPs) and a plasmid encoding the gB protein fused to the transmembrane and cytoplasmic domains of the vesicular stomatitis virus G protein (thus allowing budding from the cell surface of eVLPs incorporating gB into the lipid bilayer). This vaccine (gB-G eVLP vaccine) was found to be more immunogenic than another eVLP vaccine developed by VBI and expressing the entire gB sequence [131]. When tested in a Phase I study in adult seronegative healthy volunteers, the vaccine was immunogenic at very low doses and no severe adverse events were signaled.

*Final considerations on vaccine prevention.* The major problem related to the availability of an efficacious HCMV vaccine is due to the peculiar biological properties of this virus (as well as the other herpesviruses), such that it is not eliminated from the body following PI, but persists in a state of latency, i.e., as viral DNA associated with some IE proteins. This entails that in a lifetime, due to the occurrence of some favoring events, such as exposure to sunlight or transitory episodes of immune deficiency, viral replication can restart with final synthesis of infectious viral progeny. This is the event of virus reactivation or resumption of the replication activity by a virus strain having already infected an individual during a PI. Now, if HCMV PI would give an immune response sufficient to provide protection against subsequent episodes of reactivation, such as the case of rubella, measles and mumps, then the problem of vaccination would exist only for HCMV seronegative individuals, and a vaccine should provide an immune response comparable to that given by natural infection. However, in the case of HCMV, not only may repeated episodes of reactivation follow PI, but multiple infections by different virus strains may occur in seropositive individuals which could cause cCMV.

As a result of the above reported considerations, we can anticipate that an ideal HCMV vaccine should be able to protect against PI as well as against episodes of reactivation and reinfection. The primary endpoint of this ideal vaccine should be protection of women of childbearing age against the risk of cCMV, but the long-term endpoint would be establishment of herd immunity, also capable of protecting against both episodes of reactivation and reinfection. Whether this vaccine should be selected based on consensus sequencing of the major immunogens of multiple strains remains to be determined. However, thus far, based on current knowledge of both humoral and cellular immune responses to multiple HCMV strains, the ideal vaccine should contain: (i) gB, as an inducer of both robust antibody and T-cell responses; (ii) PC, as an inducer of the most potent NAb response; and (iii) pp65, as an inducer of the most potent T-cell response [132].

## 7. Management of Pregnancy and Counseling

According to our protocol, a pregnant woman, whenever she is referred to our center because of a suspected HCMV PI, is interviewed by an experienced medical team as early as possible. The team includes the following specialists: virologists/infectivologists/obstetricians-gynecologists. This team will manage the diagnostic test performance as well as the interpretation of the laboratory results. Then, the woman/couple will be provided with appropriate counseling according to the confirmed or excluded diagnosis of PI. Counseling is tailored by time of gestation as well as, in case of PI, by the timing of gestation at onset of infection. Advantages and limitations of treatments (HIG or valaciclovir) to prevent vertical transmission, invasive diagnostic procedures (amniocentesis) for prenatal diagnosis (when appropriate) as well as ultrasound monitoring are discussed in detail. Results of the amniocentesis are routinely available within 24 h. In case of viral detection in amniotic fluid, the chance of obtaining additional information on the fetal condition by performing cordocentesis as well as the potential antiviral treatment (if appropriate) are further discussed. Eventually, all available data are reviewed by the medical team together with the obstetrician performing the invasive procedures and the US examination. Finally, a counseling session is held. As a result, the woman/couple decides whether to continue the pregnancy or request a therapeutic abortion (up to 22 weeks of gestation). Each woman is given a written report with diagnostic conclusions and detailed instructions for neonatal testing. In a study conducted by our group on more than 700 cases of PI in pregnancy, we concluded that amniocentesis is a key option in the management of pregnancy complicated by HCMV PI. In that study, 43% of the women chose to undergo amniocentesis, while none of the women with negative amniocentesis terminated their pregnancy, and only one third of the women diagnosed with fetal infection requested termination of pregnancy [69].

## 8. Conclusions and Final Remarks

Data reported in this review are based mostly on the experience acquired by our group. Although this may represent a limitation of this review article, our studies contributed to the unraveling of the immune response to HCMV infection in view of the development of a HCMV vaccine.

Our management strategy is based on serological screening of pregnant women early during gestation or, better, pre-conceptional screening of women of childbearing age. This allows seronegative women at risk for PI to be identified and benefit from information about hygienic measures for primary prevention of PI [106]. In addition, PI can be diagnosed early and treatments for secondary prevention (i.e., prevention of virus transmission to the fetus and congenital disease [114,118]) can be proposed. Although serological screening during or before pregnancy is not universally recommended [15], we believe, on the basis of the results herein reported, that the approach here described allows a number of congenital infections as well as unnecessary pregnancy terminations to be prevented. While waiting for a HCMV vaccine, identification and counseling of pregnant women at risk for primary HCMV infection appears to be the best preventive measure.

## Data Availability

Not applicable.

## Abbreviations

HCMV, human cytomegalovirus; cCMV, congenital HCMV infection; PI, primary infection; NPI, non-primary infection; T, transmitting women; NT, non-transmitting women; AI, avidity index; gC, glycoprotein complex; TC, trimer complex; PC, pentamer complex; HELF, human embryonic lung fibroblasts; NAb, neutralizing antibodies; PDGFR-α, platelet-derived growth factor-receptor-α; IMAB, inhibition of monoclonal antibody binding; PFI, plaque-formation inhibition; LTI, leukocyte transfer inhibition; SFI, syncytium formation inhibition; mAb, monoclonal antibody; SNHL, sensorineural hearing loss; AF, amniotic fluid; US, ultrasound; DBS, dried blood spots; HIG, human immunoglobulins; RCT, randomized clinical trial; VEE, Venezuelan equine encephalitis; LCM, lymphocyte choriomeningitis; MVA, modified vaccinia Ankara; VRP, virus replicon particles; VLP, virus-like particles; IM, intramuscular; LPR, lymphoproliferative response; TCM, central memory T cells; TEM, effector memory T cells; IV, intravenous; qPCR, quantitative polymerase chain reaction; d, days.

## References

[1] Chatzakis C., Ville Y., Makrydimas G., Dinas K., Zavlanos A., Sotiriadis A. (2020). Timing of primary maternal cytomegalovirus infection and rates of vertical transmission and fetal consequences. Am. J. Obst. Gynecol..

[2] Faure-Bardon V., Magny J.-F., Parodi M., Couderc S., Garcia P., Maillotte A.-M., Benard M., Pinquier D., Astruc D., Patural H. (2019). Sequelae of congenital cytomegalovirus following maternal primary infection are limnited to those acquired in the first trimester of pregnancy. Clin. Infect. Dis..

[3] Alford C.A., Stagno S., Pass R.F., Huang E.S., Nahmias A., Dowdle W., Schinazi R. (1981). Epidemiology of cytomegalovirus. The Human Herpesviruses: An Interdisciplinary Perspective.

[4] Stagno S., Pass R.F., Dworsky M.E., Alford C.A. (1982). Maternal cytomegalovirus infection and perinatal transmission. Clin. Obstet. Gynecol..

[5] Wang C., Zhang X., Bialek S., Cannon M.J. (2011). Attribution of congenital cytomegalovirus infection to primary versus non-primary maternal infection. Clin. Infect. Dis..

[6] Cannon M.J., Schmid D.S., Hyde T.B. (2010). Review of cytomegalovirus seroprevalence and demographic characteristics associated with infection. Rev. Med. Virol..

[7] Yamamoto A.Y., Mussi-Pinhata M.M., Boppana S.B., Novak Z., Wagatsuma V.M., Oliveira P.D.F., Duarte G., Britt W.J. (2010). Human cytomegalovirus reinfection is associated with intrauterine transmission in a highly cytomegalovirus-immune maternal population. Am. J. Obstet. Gynecol..

[8] Kenneson A., Cannon M.J. (2007). Review and meta-analysis of the epidemiology of congenital cytomegalovirus (CMV) infection. Rev. Med. Virol..

[9] Dollard S.C., Grosse S.D., Ross D.S. (2007). New estimates of the prevalence of neurological and sensory sequelae and mortality asasociated with congenital cytomegalovirus infection. Rev. Med. Virol..

[10] Fowler K.B., Dahle A.J., Boppana S.B., Pass R.F. (1999). Newborn hearing screening: Will children with hearing loss caused by congenital cytomegalovirus iunfection be missed?. J. Pediatr..

[11] Boppana S.B., Pass R.F., Britt W.J., Stagno S., Alford C.A. (1992). Symptomatic congenital cytomegalovirus infection: Neonatal morbidity and mortality. Pediatr. Infect. Dis..

[12] Yamamoto A.Y., Mussi-Pinhata M.M., Isaac M.D.L., Amaral F.R., Carvalheiro C.G., Aragon D.C., da Silva Mafredi A.K., Boppana S.B., Britt W.J. (2011). Congenital cytomegalovirus infection as a cause of sensorineural hearing loss in a highly seropositive population. Pediatr. Infect. Dis..

[13] Ross S.A., Fowler K.B., Guha A., Stagno S., Britt W.J., Pass R.F., Boppana S.B. (2006). Hearing loss in children with congenital cytomegalovirus infection born to mothers with preexisting immunity. J. Pediatr..

[14] Maltezou P.G., Kourlaba G., Kourkouni Ε., Luck S., Blázquez-Gamero D., Ville Y., Lilleri D., Dimopoulou D., Karalexi M., Papaevangelou V. (2020). Maternal type of CMV infection and sequelae in infants with congenital CMV: Systematic review and meta-analysis. J. Clin. Virol..

[15] Rawlinson W.D., Boppana S.B., Fowler K.B., Kimberlin D.W., Lazzarotto T., Alain S., Daly K., Doutré S., Gibson L., Giles M.L. (2017). Congenital cytomegalovirus infection in pregnancy and the neonate: Consensus recommendations for prevention, diagnosis, and therapy. Lancet Infect. Dis..

[16] Dreher A.M., Arora N., Fowler K.B., Novak Z., Britt W.J., Boppana S.B., Ross S.A. (2014). Spectrum of disease and outcome in children with symptomatic congenital cytomegalovirus infection. J. Pediatr..

[17] Capretti M.G., Marsico C., Guidelli Guidi S., Ciardella A., Simonazzi G., Galletti S., Gabrielli L., Lazzarotto T., Faldella G. (2017). Neonatal and long-term ophthalmological findings in infants with symptomatic and asymptomatic congenital cytomegalovirus infection. J. Clin. Virol..

[18] Lopez A.S., Lanzieri T.M., Claussen A.H., Vinson S.S., Turcich M.R., Iovino I.R., Voigt R.G., Caviness A.C., Miller J.A., Williamson W.D. (2017). Intelligence and academic achievement with asymptomatic congenital cytomegalovirus infection. Pediatrics.

[19] Kabani N., Ross S.A. (2020). Congenital Cytomegalovirus Infection. J. Infect. Dis..

[20] Diener M.L., Zick C.D., McVicar S.B., Boettger J., Park A.H. (2017). Outcomes from a hearing-targeted cytomegalovirus screening program. Pediatrics.

[21] Fowler K.B., McCollister F.P., Sabo D.L., Shoup A.G., Owen K.E., Woodruff J.L., Cox E., Mohamed L.S., Choo D.I., Boppana S.B. (2017). A targeted approach for congenital cytomegalovirus screening within newborn hearing screening. Pediatrics.

[22] Gantt S., Dionne F., Kozak F.K., Goshen O., Goldfarb D.M., Park A.H., Boppana S.B., Fowler K. (2016). Cost-effectiveness of universal and targeted newborn screening for congenital cytomegalovirus infection. JAMA Pediatr..

[23] Pinninti S.G., Rodgers M.D., Novak Z., Britt W.J., Fowler K.B., Boppana S.B., Ross S.A. (2016). Clinical predictors of sensorineural hearing loss and cognitive outcome in infants with symptomatic congenital cytomegalovirus infection. Pediatr. Infect. Dis. J..

[24] de Vries L.S., Benders M.J., Groenendaal F. (2015). Progress in neonatal neurology with a focus on neuroimaging in the preterm infant. Neuropediatrics.

[25] Alarcon A., Martinez-Biarge M., Cabañas F., Hernanz A., Quero J., Garcia-Alix A. (2013). Clinical, biochemical, and neuroimaging findings predict long-term neurodevelopmental outcome in symptomatic congenital cytomegalovirus infection. J. Pediatr..

[26] de Vries J.J., van Zwet E.W., Dekker F.W., Kroes A.C., Verkerk P.H., Vossen A.C. (2013). The apparent paradox of maternal seropositivity as a risk factor for congenital cytomegalovirus infection: A population-based prediction model. Rev. Med. Virol..

[27] Lilleri D., Kabanova A., Revello M.G., Percivalle E., Sarasini A., Genini E., Sallusto F., Lanzavecchia A., Corti D., Gerna G. (2013). Fetal human cytomegalovirus transmission correlates with delayed maternal antibodies to gH/gL/pUL128-130-131 complex during primary infection. PLoS ONE.

[28] Furione M., Rognoni V., Sarasini A., Zavattoni M., Lilleri D., Gerna G., Revello M.G. (2013). Slow increase in IgG avidity correlates with prevention of human cytomegalovirus transmission to the fetus. J. Med. Virol..

[29] Pass R.F., Zhang C., Evans A., Simpson T., Andrews W., Huang M.L., Corey L., Hill J., Davis E., Flanigan C. (2009). Vaccine prevention of maternal cytomegalovirus infection. N. Engl. J. Med..

[30] Griffiths P.D., Stanton A., McCarrell E., Smith C., Osman M., Harber M., Davenport A., Jones G., Wheeler D.C., O’Beirne J. (2011). Cytomegalovirus glycoprotein-B vaccine with MF59 adjuvant in transplant recipients: A phase 2 randomised placebo-controlled trial. Lancet.

[31] Burkhardt C., Himmelein S., Britt W., Winkler T., Mach M. (2009). Glycoprotein N subtypes of human cytomegalovirus induce a strain-specific antibody response during natural infection. J. Gen. Virol..

[32] Pati S.K., Novak Z., Purser M., Arora N., Mach M., Britt W.J., Boppana S.B. (2012). Strain-specific neutralizing antibody responses against human cytomegalovirus envelope glycoprotein N. Clin. Vaccine Immunol..

[33] Kabanova A., Marcandalli J., Zhou T., Bianchi S., Baxa U., Tsybovsky Y., Lilleri D., Silacci-Fregni C., Foglierini M., Fernandez-Rodriguez B.M. (2016). Platelet-derived growth factor-α receptor is the cellular receptor for human cytomegalovirus gHgLgO trimer. Nat. Microbiol..

[34] Hahn G., Revello M.G., Patrone M., Percivalle E., Campanini G., Sarasini A., Wagner M., Gallina A., Milanesi G., Koszinowski U. (2004). Human cytomegalovirus UL131-128 genes are indispensable for virus growth in endothelial cells and virus transfer to leukocytes. J. Virol..

[35] Ryckman B.J., Rainish B.L., Chase M.C., Borton J.A., Nelson J.A., Jarvis M.A., Johnson D.C. (2008). Characterization of the human cytomegalovirus gH/gL/UL128-131 complex that mediates entry into epithelial and endothelial cells. J. Virol..

[36] Liu J., Jardetzky T.S., Chin A.L., Johnson D.C., Vanarsdall A.L. (2018). The human cytomegalovirus trimer and pentamer promote sequential steps in entry into epithelial and endothelial cells at cell surfaces and endosomes. J. Virol..

[37] Gerna G., Sarasini A., Patrone M., Percivalle E., Fiorina L., Campanini G., Gallina A., Baldanti F., Revello M.G. (2008). Human cytomegalovirus serum neutralizing antibodies block virus infection of endothelial/epithelial cells, but not fibroblasts, early during primary infection. J. Gen. Virol..

[38] Gerna G., Percivalle E., Perez L., Lanzavecchia A., Lilleri D. (2016). Monoclonal antibodies to different components of the human cytomegalovirus (HCMV) pentamer gH/gL/pUL128L and trimer gH/gL/gO as well as antibodies elicited during primary HCMV infection prevent epithelial cell syncytium formation. J. Virol..

[39] Lilleri D., Kabanova A., Lanzavecchia A., Gerna G. (2012). Antibodies against neutralization epitopes of human cytomegalovirus gH/gL/pUL128-130-131 complex and virus spreading may correlate with virus control in vivo. J. Clin. Immunol..

[40] Lopez-Vergès S., Milush J.M., Schwartz B.S., Pando M.J., Jarjoura J., York V.A., Houchins J.P., Miller S., Kang S.M., Norris P.J. (2011). Expansion of a unique CD57⁺NKG2Chi natural killer cell subset during acute human cytomegalovirus infection. Proc. Natl. Acad. Sci. USA.

[41] Noyola D.E., Fortuny C., Muntasell A., Noguera-Julian A., Muñoz-Almagro C., Alarcón A., Juncosa T., Moraru M., Vilches C., López-Botet M. (2012). Influence of congenital human cytomegalovirus infection and the NKG2C genotype on NK-cell subset distribution in children. Eur. J. Immunol..

[42] Lilleri D., Gerna G. (2017). Maternal immune correlates of protection from human cytomegalovirus transmission to the fetus after primary infection in pregnancy. Rev. Med. Virol..

[43] Chung S., Lin Y.L., Reed C., Ng C., Cheng Z.J., Malavasi F., Yang J., Quarmby V., Song A. (2014). Characterization of in vitro antibody-dependent cell-mediated cytotoxicity activity of therapeutic antibodies—Impact of effector cells. J. Immunol. Methods.

[44] Pitard V., Roumanes D., Lafarge X., Couzi L., Garrigue I., Lafon M.E., Merville P., Moreau J.F., Déchanet-Merville J. (2008). Long-term expansion of effector/memory Vdelta2-gammadelta T cells is a specific blood signature of CMV infection. Blood.

[45] Fornara C., Lilleri D., Revello M.G., Furione M., Zavattoni M., Lenta E., Gerna G. (2011). Kinetics of effector functions and phenotype of virus-specific and γδ T lymphocytes in primary human cytomegalovirus infection during pregnancy. J. Clin. Immunol..

[46] Vermijlen D., Brouwer M., Donner C., Liesnard C., Tackoen M., Van Rysselberge M., Twité N., Goldman M., Marchant A., Willems F. (2010). Human cytomegalovirus elicits fetal gammadelta T cell responses in utero. J. Exp. Med..

[47] Lozza L., Lilleri D., Percivalle E., Fornara C., Comolli G., Revello M.G., Gerna G. (2005). Simultaneous quantification of human cytomegalovirus (HCMV)-specific CD4+ and CD8+ T cells by a novel method using monocyte-derived HCMV-infected immature dendritic cells. Eur. J. Immunol..

[48] Revello M.G., Lilleri D., Zavattoni M., Furione M., Genini E., Comolli G., Gerna G. (2006). Lymphoproliferative response in primary human cytomegalovirus (HCMV) infection is delayed in HCMV transmitter mothers. J. Infect. Dis..

[49] Lilleri D., Fornara C., Furione M., Zavattoni M., Revello M.G., Gerna G. (2007). Development of human cytomegalovirus-specific T cell immunity during primary infection of pregnant women and its correlation with virus transmission to the fetus. J. Infect. Dis..

[50] Sallusto F., Lenig D., Förster R., Lipp M., Lanzavecchia A. (1999). Two subsets of memory T lymphocytes with distinct homing potentials and effector functions. Nature.

[51] Wills M.R., Carmichael A.J., Weekes M.P., Mynard K., Okecha G., Hicks R., Sissons J.G. (1999). Human virus-specific CD8+ CTL clones revert from CD45ROhigh to CD45RAhigh in vivo: CD45RAhighCD8+ T cells comprise both naive and memory cells. J. Immunol..

[52] Lilleri D., Fornara C., Revello M.G., Gerna G. (2008). Human cytomegalovirus-specific memory CD8+ and CD4+ T cell differentiation after primary infection. J. Infect. Dis..

[53] Fornara C., Furione M., Arossa A., Gerna G., Lilleri D. (2016). Comparative magnitude and kinetics of human cytomegalovirus-specific CD4⁺ and CD8⁺ T-cell responses in pregnant women with primary versus remote infection and in transmitting versus non-transmitting mothers: Its utility for dating primary infection in pregnancy. J. Med. Virol..

[54] Mele F., Fornara C., Jarrossay D., Furione M., Arossa A., Spinillo A., Lanzavecchia A., Gerna G., Sallusto F., Lilleri D. (2017). Phenotype and specificity of T cells in primary human cytomegalovirus infection during pregnancy: IL-7Rpos long-term memory phenotype is associated with protection from vertical transmission. PLoS ONE.

[55] Fornara C., Cassaniti I., Zavattoni M., Furione M., Adzasehoun K.M.G., De Silvestri A., Comolli G., Baldanti F. (2017). Human cytomegalovirus-specific memory CD4+ T-cell response and its correlation with virus transmission to the fetus in pregnant women with primary infection. Clin. Infect. Dis..

[56] Revello M.G., Lazzarotto T., Guerra B., Spinillo A., Ferrazzi E., Kustermann A., Guaschino S., Vergani P., Todros T., Frusca T. (2014). A randomized trial of hyperimmune globulin to prevent congenital cytomegalovirus. N. Engl. J. Med..

[57] Razonable R.R., Inoue N., Pinninti S.G., Boppana S.B., Lazzarotto T., Gabrielli L., Simonazzi G., Pellett P.E., Schmid D.S. (2020). Clinical diagnostic testing for human cytomegalovirus infections. J. Infect. Dis..

[58] Coppola T., Mangold J.F., Cantrell S., Permar S.R. (2019). Impact of maternal immunity on congenital cytomegalovirus birth prevalence and infant outcomes: A systematic review. Vaccines.

[59] Davis N.L., King C.C., Kourtis A.P. (2017). Cytomegalovirus infection in pregnancy. Birth Defects Res..

[60] Leruez-Ville M., Ville Y. (2017). Fetal cytomegalovirus infection. Best Pract. Res. Clin. Obstet. Gynaecol..

[61] Lazzarotto T., Varani S., Guerra B., Nicolosi A., Lanari M., Landini M.P. (2000). Prenatal indicators of congenital cytomegalovirus infection. J. Pediatr..

[62] Furione M., Sarasini A., Arossa A., Fornara C., Lilleri D., Perez L., Parea M., Zavattoni M., Spinillo A., Marone P. (2018). False human cytomegalovirus IgG-positivity at prenatal screening. J. Clin. Virol..

[63] Sarasini A., Arossa A., Zavattoni M., Fornara C., Lilleri D., Spinillo A., Baldanti F., Furione M. (2021). Pitfalls in the serological diagnosis of primary human cytomegalovirus infection in pregnancy due to different kinetics of IgM clearance and IgG avidity index maturation. Diagnostics.

[64] Revello M.G., Genini E., Gorini G., Klersy C., Piralla A., Gerna G. (2010). Comparative evaluation of eight commercial human cytomegalovirus IgG avidity assays. J. Clin. Virol..

[65] Vauloup-Fellous C., Lazzarotto T., Revello M.G., Grangeot-Keros L. (2014). Clinical evaluation of the Roche Elecsys CMV IgG Avidity assay. Eur. J. Clin. Microbiol. Infect. Dis..

[66] Sellier Y., Guilleminot T., Ville Y., Leruez-Ville M. (2015). Comparison of the LIAISON(®) CMV IgG Avidity II and the VIDAS(®) CMV IgG Avidity II assays for the diagnosis of primary infection in pregnant women. J. Clin. Virol..

[67] Delforge M.L., Eykmans J., Steensels D., Costa E., Donner C., Montesinos I. (2019). Combination of line immunoassays Mikrogen recomLine CMV IgG and recomLine CMV IgG Avidity helps to date the onset of CMV primary infection. Diagn. Microbiol. Infect. Dis..

[68] Revello M.G., Gerna G.G., Reddehase M.J. (2013). State of the Art and Trends from Cytomegalovirus Diagnostics in Cytomegaloviruses: From Molecular Pathogenesis to Intervention.

[69] Revello M.G., Fabbri E., Furione M., Zavattoni M., Lilleri D., Tassis B., Quarenghi A., Cena C., Arossa A., Montanari L. (2011). Role of prenatal diagnosis and counseling in the management of 735 pregnancies complicated by primary human cytomegalovirus infection: A 20-year experience. J. Clin. Virol..

[70] Simonazzi G., Cervi F., Zavatta A., Pellizzoni L., Guerra B., Mastroroberto M., Morselli-Labate A.M., Gabrielli L., Rizzo N., Lazzarotto T. (2017). Congenital cytomegalovirus infection: Prognostic value of maternal DNAemia at amniocentesis. Clin. Infect. Dis..

[71] Zavattoni M., Furione M., Lanzarini P., Arossa A., Rustico M., Tassis B., Piralla A., Baldanti F. (2016). Monitoring of human cytomegalovirus DNAemia during primary infection in transmitter and non-transmitter mothers. J. Clin. Virol..

[72] Revello M.G., Zavattoni M., Sarasini A., Percivalle E., Simoncini L., Gerna G. (1998). Human cytomegalovirus in blood of immunocompetent persons during primary infection: Prognostic implications for pregnancy. J. Infect. Dis..

[73] Novak Z., Ross S.A., Patro R.K., Pati S.K., Reddy M.K., Purser M., Britt W.J., Boppana S.B. (2009). Enzyme-linked immunosorbent assay method for detection of cytomegalovirus strain-specific antibody responses. Clin. Vaccine Immunol..

[74] Zavaglio F., Fiorina L., Suárez N.M., Fornara C., De Cicco M., Cirasola D., Davison A.J., Gerna G., Lilleri D. (2021). Detection of genotype-specific antibody responses to glycoproteins B and H in primary and non-primary human cytomegalovirus infections by peptide-based ELISA. Viruses.

[75] Daiminger A., Bäder U., Enders G. (2005). Pre- and periconceptional primary cytomegalovirus infection: Risk of vertical transmission and congenital disease. BJOG.

[76] Revello M.G., Zavattoni M., Furione M., Lilleri D., Gorini G., Gerna G. (2002). Diagnosis and outcome of preconceptional and periconceptional primary human cytomegalovirus infections. J. Infect. Dis..

[77] Lilleri D., Gerna G., Furione M., Zavattoni M., Spinillo A. (2016). Neutralizing and ELISA IgG antibodies to human cytomegalovirus glycoprotein complexes may help date the onset of primary infection in pregnancy. J. Clin. Virol..

[78] Penka L., Kagan K.O., Hamprecht K. (2020). Enhanced serum levels of sFlt1: Impact on materno-fetal CMV transmission. J. Clin. Med..

[79] Zavattoni M., Lombardi G., Rognoni V., Furione M., Klersy C., Stronati M., Baldanti F. (2014). Maternal, fetal, and neonatal parameters for prognosis and counseling of HCMV congenital infection. J. Med. Virol..

[80] Lazzarotto T., Guerra B., Gabrielli L., Lanari M., Landini M.P. (2011). Update on the prevention, diagnosis and management of cytomegalovirus infection during pregnancy. Clin. Microbiol. Infect..

[81] Enders M., Daiminger A., Exler S., Ertan K., Enders G., Bald R. (2017). Prenatal diagnosis of congenital cytomegalovirus infection in 115 cases: A 5 years’ single center experience. Prenat. Diagn..

[82] Bilavsky E., Pardo J., Attias J., Levy I., Magny J.F., Ville Y., Leruez-Ville M., Amir J. (2016). Clinical implications for children born with congenital cytomegalovirus infection following a negative amniocentesis. Clin. Infect. Dis..

[83] Khalil A., Sotiriadis A., Chaoui R., da Silva Costa F., D’Antonio F., Heath P.T., Jones C., Malinger G., Odibo A., Prefumo F. (2020). ISUOG Practice Guidelines: Role of ultrasound in congenital infection. Ultrasound Obstet. Gynecol..

[84] Fabbri E., Revello M.G., Furione M., Zavattoni M., Lilleri D., Tassis B., Quarenghi A., Rustico M., Nicolini U., Ferrazzi E. (2011). Prognostic markers of symptomatic congenital human cytomegalovirus infection in fetal blood. BJOG.

[85] Guerra B., Simonazzi G., Puccetti C., Lanari M., Farina A., Lazzarotto T., Rizzo N. (2008). Ultrasound prediction of symptomatic congenital cytomegalovirus infection. Am. J. Obstet. Gynecol..

[86] Picone O., Simon I., Benachi A., Brunelle F., Sonigo P. (2008). Comparison between ultrasound and magnetic resonance imaging in assessment of fetal cytomegalovirus infection. Prenat. Diagn..

[87] Farkas N., Hoffmann C., Ben-Sira L., Lev D., Schweiger A., Kidron D., Lerman-Sagie T., Malinger G. (2011). Does normal fetal brain ultrasound predict normal neurodevelopmental outcome in congenital cytomegalovirus infection?. Prenat. Diagn..

[88] Capretti M.G., Lanari M., Tani G., Ancora G., Sciutti R., Marsico C., Lazzarotto T., Gabrielli L., Guerra B., Corvaglia L. (2014). Role of cerebral ultrasound and magnetic resonance imaging in newborns with congenital cytomegalovirus infection. Brain Dev..

[89] Gerna G., Vasquez A., McCloud C.J., Chambers R.W. (1976). The immunoperoxidase technique for rapid human cytomegalovirus identification. Arch. Virol..

[90] Gleaves C.A., Smith T.F., Shuster E.A., Pearson G.R. (1984). Rapid detection of cytomegalovirus in MRC-5 cells inoculated with urine specimens by using low-speed centrifugation and monoclonal antibody to an early antigen. J. Clin. Microbiol..

[91] Gerna G., Baldanti F., Percivalle E., Zavattoni M., Campanini G., Revello M.G. (2003). Early identification of human cytomegalovirus strains by the shell vial assay is prevented by a novel amino acid substitution in UL123 IE1 gene product. J. Clin. Microbiol..

[92] Boppana S.B., Smith R.J., Stagno S., Britt W.J. (1992). Evaluation of a microtiter plate fluorescent-antibody assay for rapid detection of human cytomegalovirus infection. J. Clin. Microbiol..

[93] Balcarek K.B., Warren W., Smith R.J., Lyon M.D., Pass R.F. (1993). Neonatal screening for congenital cytomegalovirus infection by detection of virus in saliva. J. Infect. Dis..

[94] Demmler G.J., Buffone G.J., Schimbor C.M., May R.A. (1988). Detection of cytomegalovirus in urine from newborns by using polymerase chain reaction DNA amplification. J. Infect. Dis..

[95] Yamamoto A.Y., Mussi-Pinhata M.M., Marin L.J., Brito R.M., Oliveira P.F., Coelho T.B. (2006). Is saliva as reliable as urine for detection of cytomegalovirus DNA for neonatal screening of congenital CMV infection?. J. Clin. Virol..

[96] Boppana S.B., Ross S.A., Shimamura M., Palmer A.L., Ahmed A., Michaels M.G., Sánchez P.J., Bernstein D.I., Tolan R.W., Novak Z. (2011). Saliva polymerase-chain-reaction assay for cytomegalovirus screening in newborns. N. Engl. J. Med..

[97] Ross S.A., Ahmed A., Palmer A.L., Michaels M.G., Sánchez P.J., Bernstein D.I., Tolan R.W., Novak Z., Chowdhury N., Fowler K.B. (2014). Detection of congenital cytomegalovirus infection by real-time polymerase chain reaction analysis of saliva or urine specimens. J. Infect. Dis..

[98] Pinninti S.G., Ross S.A., Shimamura M., Novak Z., Palmer A.L., Ahmed A., Tolan R.W., Bernstein D.I., Michaels M.G., Sánchez P.J. (2015). Comparison of saliva PCR assay versus rapid culture for detection of congenital cytomegalovirus infection. Pediatr. Infect. Dis. J..

[99] Barbi M., Binda S., Primache V., Luraschi C., Corbetta C. (1996). Diagnosis of congenital cytomegalovirus infection by detection of viral DNA in dried blood spots. Clin. Diagn. Virol..

[100] Leruez-Ville M., Vauloup-Fellous C., Couderc S., Parat S., Ouchérif S., Castel C., Magny J.F. (2009). Retrospective diagnosis of congenital CMV infection in DBS from Guthrie cards: French experience. Arch. Pediatr..

[101] Boppana S.B., Ross S.A., Novak Z., Shimamura M., Tolan R.W., Palmer A.L., Ahmed A., Michaels M.G., Sánchez P.J., Bernstein D.I. (2010). Dried blood spot real-time polymerase chain reaction assays to screen newborns for congenital cytomegalovirus infection. JAMA.

[102] Koontz D., Dollard S., Cordovado S. (2019). Evaluation of rapid and sensitive DNA extraction methods for detection of cytomegalovirus in dried blood spots. J. Virol. Methods..

[103] Adler S.P., Finney J.W., Manganello A.M., Best A.M. (1996). Prevention of child-to-mother transmission of cytomegalovirus by changing behaviors: A randomized controlled trial. Pediatr. Infect. Dis. J..

[104] Adler S.P., Finney J.W., Manganello A.M., Best A.M. (2004). Prevention of child-to-mother transmission of cytomegalovirus among pregnant women. J. Pediatr..

[105] Vauloup-Fellous C., Picone O., Cordier A.G., Parent-du-Châtelet I., Senat M.V., Frydman R., Grangeot-Keros L. (2009). Does hygiene counseling have an impact on the rate of CMV primary infection during pregnancy? Results of a 3-year prospective study in a French hospital. J. Clin. Virol..

[106] Revello M.G., Tibaldi C., Masuelli G., Frisina V., Sacchi A., Furione M., Arossa A., Spinillo A., Klersy C., Ceccarelli M. (2015). Prevention of Primary Cytomegalovirus Infection in Pregnancy. EBioMedicine.

[107] Nigro G., Adler S.P., La Torre R., Best A.M., Congenital Cytomegalovirus Collaborating Group (2005). Passive immunization during pregnancy for congenital cytomegalovirus infection. N. Engl. J. Med..

[108] Buxmann H., Stackelberg O.M., Schlößer R.L., Enders G., Gonser M., Meyer-Wittkopf M., Hamprecht K., Enders M. (2012). Use of cytomegalovirus hyperimmunoglobulin for prevention of congenital cytomegalovirus disease: A retrospective analysis. J. Perinat. Med..

[109] Enders G., Daiminger A., Bäder U., Exler S., Enders M. (2011). Intrauterine transmission and clinical outcome of 248 pregnancies with primary cytomegalovirus infection in relation to gestational age. J. Clin. Virol..

[110] Bodéus M., Kabamba-Mukadi B., Zech F., Hubinont C., Bernard P., Goubau P. (2010). Human cytomegalovirus in utero transmission: Follow-up of 524 maternal seroconversions. J. Clin. Virol..

[111] Hughes B. (2019). LB17. Randomized Trial to Prevent Congenital Cytomegalovirus (CMV) Open Forum Infect. Dis..

[112] Hamprecht K., Kagan K.O., Goelz R. (2014). Hyperimmune globulin to prevent congenital CMV infection. N. Engl. J. Med..

[113] Kagan K.O., Enders M., Schampera M.S., Baeumel E., Hoopmann M., Geipel A., Berg C., Goelz R., De Catte L., Wallwiener D. (2019). Prevention of maternal-fetal transmission of cytomegalovirus after primary maternal infection in the first trimester by biweekly hyperimmunoglobulin administration. Ultrasound Obstet. Gynecol..

[114] Kagan K.O., Enders M., Hoopmann M., Geipel A., Simonini C., Berg C., Gottschalk I., Faschingbauer F., Schneider M.O., Ganzenmueller T. (2021). Outcome of pregnancies with recent primary cytomegalovirus infection in first trimester treated with hyperimmunoglobulin: Observational study. Ultrasound Obstet. Gynecol..

[115] Bartlett A.W., Hamilton S.T., Shand A.W., Rawlinson W.D. (2020). Fetal therapies for cytomegalovirus: What we tell prospective parents. Prenat. Diagn..

[116] Jacquemard F., Yamamoto M., Costa J.M., Romand S., Jaqz-Aigrain E., Dejean A., Daffos F., Ville Y. (2007). Maternal administration of valaciclovir in symptomatic intrauterine cytomegalovirus infection. BJOG.

[117] Pasternak B., Hviid A. (2010). Use of acyclovir, valacyclovir, and famciclovir in the first trimester of pregnancy and the risk of birth defects. JAMA.

[118] Shahar-Nissan K., Pardo J., Peled O., Krause I., Bilavsky E., Wiznitzer A., Hadar E., Amir J. (2020). Valaciclovir to prevent vertical transmission of cytomegalovirus after maternal primary infection during pregnancy: A randomised, double-blind, placebo-controlled trial. Lancet.

[119] Gazzetta Ufficiale della Repubblica Italiana n.322.

[120] Roxby A.C., Atkinson C., Asbjörnsdóttir K., Farquhar C., Kiarie J.N., Drake A.L., Wald A., Boeckh M., Richardson B., Emery V. (2014). Maternal valacyclovir and infant cytomegalovirus acquisition: A randomized controlled trial among HIV-infected women. PLoS ONE.

[121] Plotkin S.A. (1975). Vaccination against herpes group viruses. Pediatrics.

[122] Elek S.D., Stern H. (1974). Letter: Vaccination against cytomegalovirus?. Lancet.

[123] Fu T.M., Wang D., Freed D.C., Tang A., Li F., He X., Cole S., Dubey S., Finnefrock A.C., ter Meulen J. (2012). Restoration of viral epithelial tropism improves immunogenicity in rabbits and rhesus macaques for a whole virion vaccine of human cytomegalovirus. Vaccine.

[124] Kemble G., Duke G., Winter R., Spaete R. (1996). Defined large-scale alterations of the human cytomegalovirus genome constructed by cotransfection of overlapping cosmids. J. Virol..

[125] Wang D., Freed D.C., He X., Li F., Tang A., Cox K.S., Dubey S.A., Cole S., Medi M.B., Liu Y. (2016). A replication-defective human cytomegalovirus vaccine for prevention of congenital infection. Sci. Transl. Med..

[126] Adler S.P., Lewis N., Conlon A., Christiansen M.P., Al-Ibrahim M., Rupp R., Fu T.M., Bautista O., Tang H., Wang D. (2019). Phase 1 Clinical Trial of a Conditionally Replication-Defective Human Cytomegalovirus (CMV) Vaccine in CMV-Seronegative Subjects. J. Infect. Dis..

[127] Pass R.F., Duliegè A.M., Boppana S., Sekulovich R., Percell S., Britt W., Burke R.L. (1999). A subunit cytomegalovirus vaccine based on recombinant envelope glycoprotein B and a new adjuvant. J. Infect. Dis..

[128] Sabbaj S., Pass R.F., Goepfert P.A., Pichon S. (2011). Glycoprotein B vaccine is capable of boosting both antibody and CD4 T-cell responses to cytomegalovirus in chronically infected women. J. Infect. Dis..

[129] Bernstein D.I., Munoz F.M., Callahan S.T., Rupp R., Wootton S.H., Edwards K.M., Turley C.B., Stanberry L.R., Patel S.M., Mcneal M.M. (2016). Safety and efficacy of a cytomegalovirus glycoprotein B (gB) vaccine in adolescent girls: A randomized clinical trial. Vaccine.

[130] John S., Yuzhakov O., Woods A., Deterling J., Hassett K., Shaw C.A., Ciaramella G. (2018). Multi-antigenic human cytomegalovirus mRNA vaccines that elicit potent humoral and cell-mediated immunity. Vaccine.

[131] Kirchmeier M., Fluckiger A.C., Soare C., Bozic J., Ontsouka B., Ahmed T., Diress A., Pereira L., Schödel F., Plotkin S. (2014). Enveloped virus-like particle expression of human cytomegalovirus glycoprotein B antigen induces antibodies with potent and broad neutralizing activity. Clin. Vaccine Immunol..

[132] Gerna G., Lilleri D. (2019). Human cytomegalovirus (HCMV) infection/re-infection: Development of a protective HCMV vaccine. New Microbiol..

